# Nasu-Hakola Disease Presenting as Rapidly Progressive Dementia With Seizures: A TREM2 Mutation Case Without Skeletal Involvement

**DOI:** 10.7759/cureus.98664

**Published:** 2025-12-07

**Authors:** Zubair Sarkar, Md Mahmood Alam, Ayushi Chaudhari

**Affiliations:** 1 Medicine, Naraina Medical College and Research Centre, Kanpur, IND

**Keywords:** dementia with seizures, early-onset dementia, microglial dysfunction, nasu-hakola disease, trem2 gene mutation

## Abstract

Nasu-Hakola disease (NHD) is a rare autosomal recessive disorder caused by mutations in the TYROBP or TREM2 genes. It is characterised by the unique combination of neurological and skeletal manifestations. However, isolated neurological variants without bone involvement have been described. A 38-year-old woman presented with a two-year history of progressive cognitive decline, reduced speech output, inappropriate emotional behaviour, recurrent generalised tonic-clonic seizures, gait unsteadiness, and loss of self-care abilities. Neurological examination revealed Parkinsonian features, cerebellar signs, brisk reflexes, and frontal release signs. Laboratory investigations for metabolic, autoimmune, and infectious aetiology were normal. Brain MRI demonstrated diffuse cortical atrophy, periventricular T2/fluid-attenuated inversion recovery (FLAIR) hyperintensities, and bilateral globus pallidus calcification. Skeletal radiographs showed generalised osteopenia without cystic lesions. Clinical exome sequencing detected a homozygous c.371T>G mutation in the TREM2 gene (exon 2), confirming the diagnosis of Nasu-Hakola disease. NHD is typically described to progress in four stages, starting from a latent asymptomatic phase to sequential bone and neurological involvement. While bone involvement is a classical feature, this case emphasises isolated neurological involvement associated with TREM2 mutations. Furthermore, this case highlights the importance of neuroimaging combined with next-generation sequencing in patients with early-onset dementia with atypical features like generalised seizures and Parkinsonism. Nasu-Hakola disease should be considered in the differential diagnosis of presenile dementia, even in the absence of skeletal manifestations. Early recognition through genetic testing facilitates accurate diagnosis, appropriate counselling, and avoidance of unnecessary investigations.

## Introduction

Nasu-Hakola disease (NHD), also known as polycystic lipomembranous osteodysplasia with sclerosing leukoencephalopathy (PLOSL), is an uncommon hereditary disorder that is transmitted in an autosomal recessive manner. Reported cases have been primarily concentrated in Japanese and Finnish cohorts, with only a few cases reported from the Indian subcontinent [1]. The hallmark of this disease is an unusual combination of early-onset dementia with skeletal involvement in the form of bone cysts and pathological fractures [2]. However, this case emphasises the need to consider NHD in cases of early-onset dementia with or without skeletal involvement in appropriate clinical scenarios.

## Case presentation

We describe the case of a 38-year-old homemaker who presented with a two-year history of progressive cognitive decline, markedly reduced word output, inappropriate smiling, behavioural disturbances, multiple episodes of seizures (generalised tonic-clonic seizures (GTCS)), difficulty in walking, and inability to care for herself. Her parents were not consanguineous, and there was no family history of similar illness.

The progression was quite rapid, involving multiple cognitive domains within a year of onset. Cognitive assessment with Mini-Mental State Examination (MMSE) [3] and neuropsychological testing could not be done due to advanced inattention and dementia. Upon further examination, she exhibited Parkinsonian features, including axial and limb rigidity, reduced facial expressions, and cerebellar signs, including limb and gait ataxia. Deep tendon reflexes were brisk, and plantar reflexes were bilateral extensor. Frontal release signs, including glabellar tap and palmomental reflex, were present.

**Figure 1:**
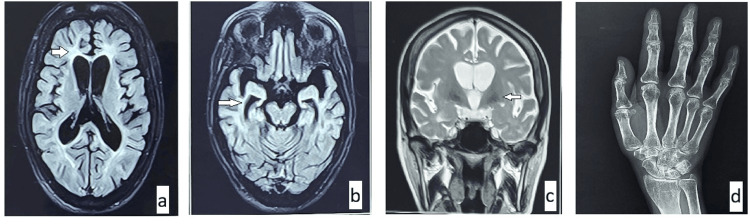
MRI brain and X-ray findings. (a) and (b) FLAIR images showing periventricular white matter hyperintensity and diffuse cortical atrophy. (c) T2 image showing bilateral globus pallidus calcification. (d) X-ray of the right hand demonstrating periarticular osteopenia. FLAIR: fluid-attenuated inversion recovery.

The patient was extensively evaluated for reversible causes. All investigations, including biochemical workup, autoimmune markers, and cerebrospinal fluid analysis, were unremarkable. These investigations comprehensively ruled out metabolic, endocrine, nutritional and autoimmune causes of dementia. MRI brain scans revealed diffuse cortical atrophy, along with widespread T2/fluid-attenuated inversion recovery (FLAIR) hyperintensities involving the periventricular white matter and calcification in the bilateral globus pallidus (Figures 1a-1d). X-ray of both hands showed generalised osteopenia without visible cystic lesions (Figure 1e). Clinical exome sequencing identified a homozygous c.371T>G mutation in the TREM2 gene located on exon 2, confirming the diagnosis of NHD.

## Discussion

NHD was first recognised in the 1970s when it was independently described by Nasu and Hakola [2]. This autosomal recessive inherited disorder is characterised by progressive dementia and repeated pathological fractures [2]. Pathogen mutations in either TRYOBP or TREM2 genes underlie NHD. The TREM2-TRYOBP protein complex is involved in the regulation of differentiation and function of osteoclasts [4]. Within the central nervous system, the same complex is primarily expressed in microglia and plays a role in regulating their survival and phagocytic activity [5]. The dysfunction of this signalling pathway leads to impaired clearance of apoptotic neurons and increased proinflammatory responses, causing brain injury and neurodegeneration [6]. NHD is therefore increasingly recognised as a prototypical disorder arising from primary microglial dysfunction [7].

Hakola described the clinical course of NHD in four stages: an initial asymptomatic or latent phase, followed by a skeletal phase marked by pathological fractures and bone cysts, and subsequent early and late neuropsychiatric phases characterised by progressive cognitive and behavioural decline [8]. Neurological symptoms usually start in the third or fourth decade, starting with behaviour change and memory disturbances, like features observed in cases of frontotemporal dementia. Other symptoms include gait disturbances, emotional dysregulation, apathy, and seizures. These symptoms are generally progressive and lead to severe impairment of memory and function. However, presentations vary considerably, and the disease has been shown to affect only the central nervous system, without bone involvement. Patients with TREM2 mutations have been specifically reported with pure neurological involvement [9]. The differential diagnosis included causes of early onset and rapidly progressive dementia, including those with autoimmune aetiology and familial forms of frontotemporal dementia and Alzheimer’s disease.

The absence of family history, early onset in the fourth decade, and a prominent association with recurrent seizures, along with characteristic neuroimaging features such as diffuse cortical atrophy, periventricular hyperintensities, and bilateral basal ganglia calcifications, led us to consider NHD in the differential diagnosis. After ruling out all the reversible causes with relevant investigations, a clinical exome sequencing was ordered, which confirmed the TREM2 mutation and, consequently, the diagnosis of NHD.

## Conclusions

This case emphasises the need to consider NHD as a differential diagnosis in cases of presenile dementia, especially when associated with atypical symptoms like generalised seizures and Parkinsonism. Furthermore, the absence of skeletal manifestations shall not preclude the diagnosis of NHD. Early recognition through genetic testing facilitates accurate diagnosis, appropriate counselling, and avoidance of unnecessary investigations.
